# Prognostic nomogram for predicting 5-year overall survival in Chinese patients with high-grade osteosarcoma

**DOI:** 10.1038/s41598-021-97090-0

**Published:** 2021-09-06

**Authors:** Zhihong Yao, Zunxian Tan, Jifei Yang, Yihao Yang, Cao Wang, Jiaxiang Chen, Yanan Zhu, Tiying Wang, Lei Han, Lin Zhu, Zuozhang Yang

**Affiliations:** 1 Bone and Soft Tissue Tumours Research Centre of Yunnan Province, Department of Orthopaedics, The Third Affiliated Hospital of Kunming Medical University (Yunnan Cancer Hospital), Kunming, 650118 Yunnan 119 Kunzhou Road, China; 2 Independent Investigator, No. 22 Xi guan North Road, Kaifeng, 475000 Henan China

**Keywords:** Bone cancer, Cancer therapy

## Abstract

This study aimed to construct a widely accepted prognostic nomogram in Chinese high-grade osteosarcoma (HOS) patients aged ≤ 30 years to provide insight into predicting 5-year overall survival (OS)*.* Data from 503 consecutive HOS patients at our centre between 12/2012 and 05/2019 were retrospectively collected. Eighty-four clinical features and routine laboratory haematological and biochemical testing indicators of each patient at the time of diagnosis were collected. A prognostic nomogram model for predicting OS was constructed based on the Cox proportional hazards model. The performance was assessed by the concordance index (C-index), receiver operating characteristic curve and calibration curve. The utility was evaluated by decision curve analysis. The 5-year OS was 52.1% and 2.6% for the nonmetastatic and metastatic patients, respectively. The nomogram included nine important variables based on a multivariate analysis: tumour stage, surgical type, metastasis, preoperative neoadjuvant chemotherapy cycle, postoperative metastasis time, mean corpuscular volume, tumour-specific growth factor, gamma-glutamyl transferase and creatinine. The calibration curve showed that the nomogram was able to predict 5-year OS accurately. The C-index of the nomogram for OS prediction was 0.795 (range, 0.703–0.887). Moreover, the decision curve analysis curve also demonstrated the clinical benefit of this model. The nomogram provides an individualized risk estimate of the 5-year OS in patients with HOS aged ≤ 30 years in a Chinese population-based cohort.

## Introduction

In the past several decades, high-grade osteosarcoma (HOS) has always been the most common primary bone malignancy, typically affecting children and adolescents, and its clinical prognosis is poor^1,2^. Thanks to the era of neoadjuvant chemotherapy and limb salvage, the overall survival (OS) of non-metastatic HOS has increased to approximately 70% from less than 20%. However, these outcome data cannot be generalized to all patients. For patients with distant metastasis or local recurrence, the OS remains less than 20%^3^. Recent reports have suggested that approximately 40% of patients have metastases at initial diagnosis^4^. Among those who do not have metastasis at initial diagnosis, 20–30% of patients will develop distant metastasis within a year, even if they have already been treated. There is undoubtedly an urgent need to accurately forecast the risk of death to guide clinical therapy and improve the curative treatment and survival rate of HOS patients.

Studies of the prognostic-associated risk factors for HOS have developed rapidly, and many kinds of prognostic factors have been reported, such as sex^5^, primary tumour site, tumour size^6^, tumour stage^7^, age at diagnosis^8^, pathologic fracture^9^, chemotherapy regimen and response to chemotherapy^10^, elevated serum lactate dehydrogenase (LDH) level, elevated alkaline phosphatase value (ALP) level^11^, presence of distant metastasis^12^, elevated serum C-reactive protein (CRP) level, and genetic factors^13^. Although prognostic factors and outcomes have been studied extensively in other countries, the amount of evidence-based information about Chinese HOS patients remains limited and controversial. To our knowledge, only five articles have reported nomogram models of prognosis in Chinese population-based patients with osteosarcoma^4^. However, they mainly focused on a single factor, either inflammatory factors^14^ or radiomics factors^15^. In practice, a single factor is unsatisfactory in predicting the outcome of a disease. Most importantly, there was a lack of robust information on survival and prognosis. Therefore, one of the purposes of this study was to report the survival outcomes and prognostic factors significantly associated with the OS of HOS in a Chinese population-based cohort.

In recent years, routine laboratory parameters have been confirmed as valuable prognostic factors in various types of tumours^16^ because clinicians can easily monitor their dynamic changes in the clinic. Serum LDH is a typical inflammatory marker associated with tumour burden. High levels of LDH may lead to excess production of lactic acid and acidification of the extracellular water space, helping to improve the invasion ability of cancer cells. Recent studies have confirmed that in a variety of cancer types, elevated serum LDH levels before treatment are associated with poor prognosis in patients^17^. Mei et al.^18^ confirmed through a meta-analysis of 24,536 advanced tumour patients included in 66 studies that an elevated neutrophil-to-lymphocyte ratio (NLR) in peripheral blood before treatment was directly correlated with shortened PFS and OS in advanced tumour patients. Dell et al.^19^ confirmed the prognostic role of the NLR in metastatic colorectal cancer through a multi-centre phase III trial conducted in 413 patients. In the clinic, nomograms are widely used in the initial diagnosis, tumour staging, detection of recurrence and assessment of clinical prognosis in many kinds of cancer^20^. To make our findings more credible, we intend to develop a nomogram to foresee the death risk of HOS based on Chinese patients.

Therefore, the aim of this study was to clarify the outcome and reliable clinical prognostic factors for the Chinese population with HOS to help surgeons predict prognosis and guide management for HOS patients. To our knowledge, this is the first study to use a nomogram model to predict OS in the Chinese population based on more comprehensive data from conventional laboratory analyses.

## Results

### Characteristics of patients

On the basis of the entry criteria, 123 patients with HOS from December 2012 to May 2019 were included and analysed. Of the 123 patients, 89 patients (72.4%) were male, for male: female ratio of 2.62:1. The median age at the first diagnosis was 16.6 years (range 3–30 years). The proportion of primary lesions was as follows: femur 51.2%, tibia 31.7%, fibula 6.5% and humerus 4.1%. Fourteen patients suffered fracture at the first diagnosis. Forty-seven patients (38.2%) did not complete the preoperative chemotherapy. Seventy-five patients (61%) had metastatic disease, and the mean metastasis time was 8.3 months, among whom 51 (68%) had double pulmonary metastases, 15 (20%) had single pulmonary metastases, and 9 (12%) had bone metastases or lymph node metastases. Among them, 26 (34.7%) patients had metastases at the time of initial diagnosis, and 47 (62.7%) patients had metastases within 2 years. The baseline clinical characteristics of HOS patients in the alive group and death group are presented in Table 1, which lists the baseline demographics and the factors with significant differences, and Supplementary Table S1 lists the factors with continuous variables. Tumour stage (*p* < 0.001), metastasis (*p* < 0.001), complete preoperative chemotherapy cycle (*p* = 0.003), elevated serum TSGF level (*p* = 0.004), elevated serum LDH level (*p* = 0.001), elevated serum Mg (*p* = 0.001), elevated serum ALP (*p* = 0.02), MCHC (*p* = 0.011) and RDW. SD (*p* = 0.039) was associated with the survival of Chinese HOS patients. The baseline demographics and clinical characteristics of HOS patients in the metastasis group and non-metastasis group are presented in Supplementary Table S2. Tumour stage (*p* < 0.001), elevated ALP (*p* = 0.006), and elevated LDH (*p* = 0.004) were associated with the presence of metastases.

**Table 1:** Baseline demographics and clinical characteristics of HOS patients in the alive group and death group.

Variables	Alive group (N = 25)	Death group (N = 98)	Total	*p*
**Age (years)**				0.596
≥ 18	16 (64)	57 (58.16)	73 (59.36)	
< 18	9 (36)	41 (41.84)	50 (27.64)	
**Gender**				0.648
Male	19 (76)	70 (71.43)	89 (72.36)	
Female	6 (24)	28 (28.57)	34 (27.64)	
**Primary tumour site**				0.953
Femur	14 (56)	49 (50)	63 (51.22)	
Fibula	1 (4)	7 (7.14)	8 (6.50)	
Humerus	1 (4)	4 (4.08)	5 (4.07)	
Tibia	7 (28)	32 (32.65)	39 (31.71)	
Other	2 (8)	6 (6.12)	8 (6.50)	
**Tumour stage**				< 0.001
IIA	8 (32)	7 (7.14)	15 (12.20)	
IIB	17 (68)	68 (69.39)	85 (69.11)	
III	0 (0)	23 (23.47)	23 (18.70)	
**Tumour size**				0.069
< 10	15 (60)	39 (39.80)	54 (43.90)	
≥ 10	10 (40)	59 (60.20)	69 (56.10)	
**Pathological fracture**				0.913
Yes	3 (12)	11 (11.22)	14 (11.38)	
No	22 (88)	87 (88.78)	109 (88.61)	
**Preoperative neoadjuvant chemotherapy**				0.540
ADM + NDP	6 (24)	19 (19.39)	25 (20.33)	
ADM + DDP + MTX + VCR	2 (8)	12 (12.24)	14 (11.38)	
ADM + DDP + MTX + VCR + IFO	17 (68)	61 (62.24)	78 (63.41)	
No	0 (0)	6 (6.12)	6 (4.88)	
**Type of surgical**				0.182
Amputation	12 (48)	40 (40.82)	52 (42.28)	
Limb salvage	13 (52)	46 (46.94)	59 (47.97)	
No	0 (0)	12 (12.24)	12 (9.76)	
**Metastasis**				< 0.001
No	23 (92)	25 (25.51)	48 (39.02)	
Yes	2 (8)	73 (74.49)	75 (60.98)	
**Recurrence**				0.332
Yes	1 (4)	10 (10.20)	11 (8.95)	
No	24 (96)	88 (89.80)	112 (91.05)	
**Complete the preoperative chemotherapy cycle**				0.003
Yes	22 (88)	54 (55.1)	76 (61.79)	
No	3 (22)	44 (44.9)	47 (38.21)	
**TSGF group (cut off = 54 ± 3.0, U/mL)**				0.004
Normal	19 (95)	48 (61.5)	67 (68.36)	
Elevated	1 (5)	30 (38.5)	31 (31.63)	
**GGT (cut off = 113 ± 1.8, U/L)**				0.286
Normal	25 (100)	65 (95.6)	90 (96.8)	
Elevated	0 (0)	3 (4.4)	3 (3.2)	
**MCV (cut off = 90.1 ± 2.7, fL)**				0.086
Normal	23 (92.0)	75 (76.5)	98 (79.7)	
Elevated	2 (8.0)	23 (23.5)	25 (20.3)	
**CREA (cut off = 68 ± 3.1, μmol/L)**				0.139
Normal	11 (44)	64 (65.3)	75 (60.98)	
Elevated	14 (56)	34 (34.7)	48 (39.02)	
**LDH (cut off = 185 ± 3.8, U/L)**				0.001
Normal	16 (64)	29 (29.6)	45 (36.6)	
Elevated	9 (36)	69 (70.4)	78 (63.4)	
**ALP (cut off = 343 ± 2.6, U/L)**				0.02
Normal	23 (92)	68 (69.4)	91 (74)	
Elevated	2 (8)	30 (30.6)	32 (26)	

The cut-off value was determined based on receiver operating characteristic curve analysis (ROC).

### Surgical treatment and response to preoperative chemotherapy

Among those patients, 111 patients (90.2%) received excision of the tumour in situ, of whom 109 patients (98.2%) received preoperative neoadjuvant chemotherapy. Among the 123 patients, 59 patients (48%) underwent limb salvage surgery, while 52 patients (42.3%) underwent amputation surgery. A total of 9.8% (12/123) of patients did not undergo any surgical intervention and did not complete the course of chemotherapy. After surgery, 11 (9.9%) patients experienced local relapse, and the mean recurrence time was 15 months. Of these patients, 9 (81.8%) underwent limb salvage surgery, and 9 (81.8%) had metastatic disease.

### Overall survival

The median follow-up was 28.4 months (range, 1–81 months). Until the time of last follow-up, 98 patients (79.7%) died due to osteosarcoma during the period. The 5-year overall survival rate was 20.3% (25/123). The median survival time was 15 months (range, 1–58 months). Sixty patients (48.8%) received complete chemotherapy and surgery, and the 5-year OS was 26.7%. In our data, 75 patients developed distant metastasis, with a 5-year OS of 2.6%. Among the metastasis patients, 2 patients were still alive because the metastasis site was solitary lung or bone, and all received complete preoperative chemotherapy. There were 48 non-metastasis patients with a 5-year survival rate of 52.1%. There were significant differences in the 5-year survival rate between the non-metastatic patient group and metastatic patient group. The patients who did not complete preoperative chemotherapy had significantly poorer survival. There were no significant differences in the survival rate in those factors: age (≥ 18 vs < 18, *p* = 0.596), sex (male vs female, *p* = 0.648), ethnicity (Han vs Minority, *p* = 0.618), primary tumour site (femur vs fibula vs humerus vs tibia vs other, *p* = 0.953), tumour size (< 10 vs ≥ 10, *p* = 0.069), pathological fracture (yes vs no, *p* = 0.913), or preoperative neoadjuvant chemotherapy regimen (ADM + NDP vs ADM + DDP + MTX + VCR vs ADM + DDP + MTX + VCR + IFO vs No, *p* = 0.540). Type of surgery (amputation vs limb salvage vs No, *p* = 0.182). The potential prognostic factors for 5-year OS were as follows: tumour stage (IIA vs IIB vs III, *p* < 0.001), metastasis (yes vs no, *p* < 0.001), complete preoperative chemotherapy cycle (yes vs no, *p* = 0.003), TSGF group (normal vs elevated, *p* = 0.004), LDH group (normal vs elevated, *p* = 0.001) and ALP group (normal vs elevated, *p* = 0.02).

### Multivariate Cox regression models

**Figure 1:**
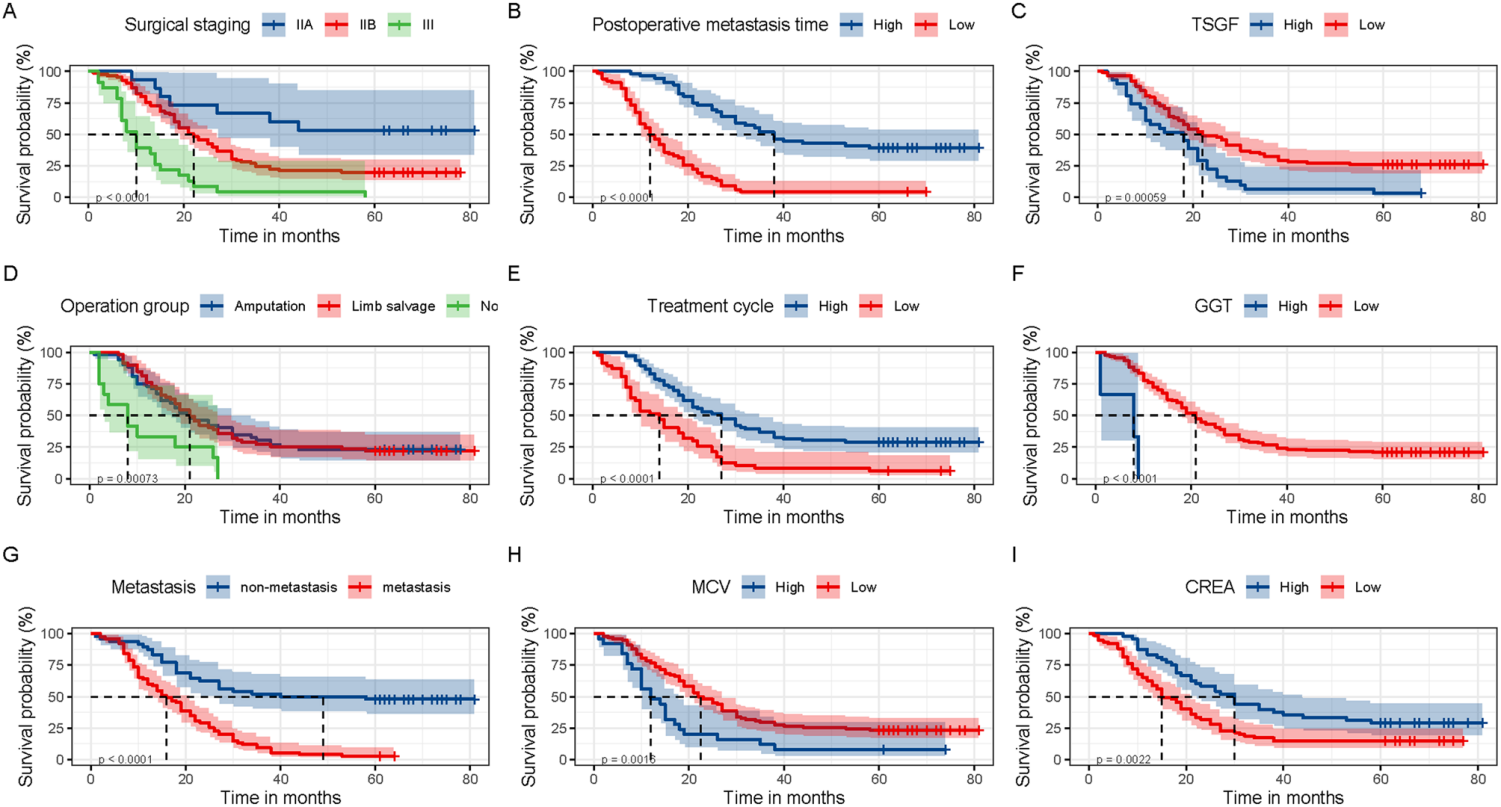
Kaplan–Meier survival curves of tumour-related survival for significant factors in univariate and multivariate Cox proportional regression models. Kaplan–Meier curves of tumour-related survival according to the surgical staging (**A**), postoperative metastasis time (**B**), TSGF group (**C**), operation group (**D**), treatment cycle (**E**), GGT group (**F**), metastasis group (**G**), MCV group (**H**), and CREA group (**I**). A log-rank *p* value < 0.05 was considered statistically significant. *MCV* mean corpuscular volume, *TSGF* tumour-specific growth factor; treatment cycle, cycles of preoperative neoadjuvant chemotherapy, *GGT* glutamyl transferase, *CREA* creatinine.

The results of univariate and multivariate Cox regression analysis for overall survival are shown in Table 2, which lists the risk factors with significant differences, and Supplementary Table S3 lists the factors with no significant differences in univariate analysis. Univariate analyses suggested that tumour stage (*p* < 0.001), metastasis (*p* < 0.001), surgical type (*p* = 0.049), complete treatment cycle (*p* < 0.001), postoperative metastasis time (*p* = 0.008), ALP (*p* = 0.042), LDH (*p* = 0.006), TSGF (*p* = 0.002), CA-724 (*p* = 0.011), GGT (*p* = 0.014), CREA (*p* = 0.047), serum Mg (*p* < 0.001), RDW-SD (*p* = 0.009), MCHC (*p* = 0.008), MCV (*p* = 0.031), and BASO (*p* < 0.001) were significantly associated with OS. The Kaplan–Meier analyses and log-rank tests also been confirmed the results from the univariate Cox analysis. According to the Kaplan–Meier methods, the surgical staging (Fig. 1A), postoperative metastasis time (Fig. 1B), TSGF group (Fig. 1C), operation group (Fig. 1D), treatment cycle (Fig. 1E), GGT group (Fig. 1F), metastasis group (Fig. 1G), MCV group (Fig. 1H), and CREA group (Fig. 1I) were related to OS in patients with HOS. We included the variables with *p* values less than 0.05 in the multivariate Cox regression analyses. We confirmed that the following factors were independently and significantly correlated with improved OS for HOS: tumour stage (*p* < 0.001), metastasis (HR = 0.210, 95% CI 0.082–0.541, *p* < 0.001), complete treatment cycle (HR = 17.890, 95% CI 4.874–65.667, *p* < 0.001), postoperative metastasis time (*p* < 0.001), TSGF (HR = 1.167, 95% CI 1.090–1.249, *p* < 0.001), CA-724 (HR = 1.220, 95% CI 1.089–1.366, *p* < 0.001), GGT (HR = 0.927, 95% CI 0.884–0.972, *p* = 0.002), RDW-SD (HR = 1.230, 95% CI 1.066–1.420, *p* = 0.005), and MCV (HR = 1.202, 95% CI 1.043–1.385, *p* = 0.011) were independent prognostic factors for HOS.

**Table 2:** Univariate and multivariate Cox proportional hazards regression analysis for the risk factors associated with the survival of HOS patients.

Variables	Univariate analysis		Multivariate analysis	
	HR (95% CI)	*p*	HR (95% CI)	*p*
Age (years)	0.934 (0.625–1.396)	0.739		
Gender (male vs female)	0.772 (0.498–1.197)	0.247		
Race (Han vs minority)	1.127 (0.616–2.063)	0.698		
BMI (< 22 vs ≥ 22)	0.941 (0.525–1.687)	0.837		
Primary tumour site (femur vs fibula vs humerus vs tibia)	0.982 (0.861–1.120)	0.790		
Tumour stage (IIA vs IIB vs III)	0.130 (0.055–0.307)	< 0.001		< 0.001
Tumour size (< 10 vs ≥ 10)	0.728 (0.486–1.092)	0.125		
Pathological fracture (yes vs no)	1.087 (0.580–2.036)	0.795		
Preoperative neoadjuvant chemotherapy (AP vs AP + MTX + VCR vs AP + MTX + VCR + IFO)	1.062 (0.837–1.346)	0.621		
Recurrence (yes vs no)	1.315 (0.682–2.534)	0.413		
First visit interval transfer time	0.490 (0.030–7.949)	0.616		
Postoperative metastasis time		0.008		< 0.001
Metastasis (yes vs no)	0.277 (0.173–0.445)	< 0.001	0.210 (0.082–0.541)	< 0.001
Type of surgical (amputation vs limb salvage)	1.406 (1.002–1.974)	0.049	1.726 (0.884–3.370)	0.110
Complete the preoperative chemotherapy cycle (yes vs no)	2.488 (1.659–3.733)	< 0.001	17.89(4.874–65.667)	< 0.001
ALP, U/L	1.000 (1.000–1.000)	0.042	1.000 (0.999–1.000)	0.484
LDH, U/L	1.001 (1.000–1.001)	0.006	1.002 (0.999–1.006)	0.192
TSGF, U/mL	1.034 (1.013–1.055)	0.002	1.167 (1.090–1.249)	< 0.001
CA724, kU/L	1.088 (1.020–1.162)	0.011	1.220 (1.089–1.366)	0.001
Serum Mg, mmol/L	95.601 (25.288–33,150.69)	< 0.001	0.112 (0.000–50,985)	0.742
GGT, U/L	1.006 (1.001–1.011)	0.014	0.927 (0.884–0.972)	0.002
CREA, μmol/L	0.988 (0.976–1.000)	0.047	1.006 (0.970–1.042)	0.759
RDW-SD, fL	1.091 (1.022–1.166)	0.009	1.230 (1.066–1.420)	0.005
MCHC, g/L	0.980 (0.966–0.995)	0.008	1.002 (0.964–1.043)	0.902
MCV, fL	1.049 (1.004–1.095)	0.031	1.202 (1.043–1.385)	0.011
BASO, 10^9^/L	44,471,424.37 (9677.90–2.044)	< 0.001		

*HR* hazard ratio, *CI* confidence interval.

### Nomogram development and internal validation

**Figure 2:**
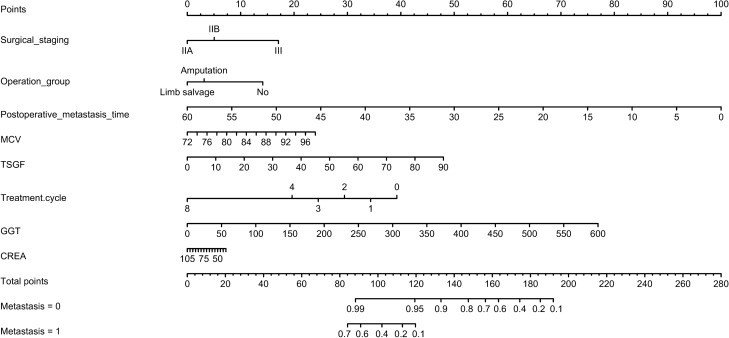
Nomogram of clinical and laboratory risk factors for predicting the probability tumour-related survival of HOS patients at 5 years. *Note*: The scores of each index were added to obtain the total score, and a vertical line was drawn to the total points to obtain the probability of death. To use, find the prognostic factor axis, then draw a vertical line upwards to the “points” axis to determine the score of this factor. This procedure was conducted again to score the other prognostic factors. The scores were summed, and the total number was located on the line “Total Points”. A vertical line was drawn downwards from the total points to determine the tumour-related survival prediction at the intersection with the 5-year survival probability for the non-metastasis patients (metastasis = 0) and metastasis patients (metastasis = 1). *MCV,* mean corpuscular volume; *TSGF,* tumour-specific growth factor; treatment cycle, cycles of preoperative neoadjuvant chemotherapy; *GGT,* glutamyl transferase; *CREA,* creatinine.

**Figure 3:**
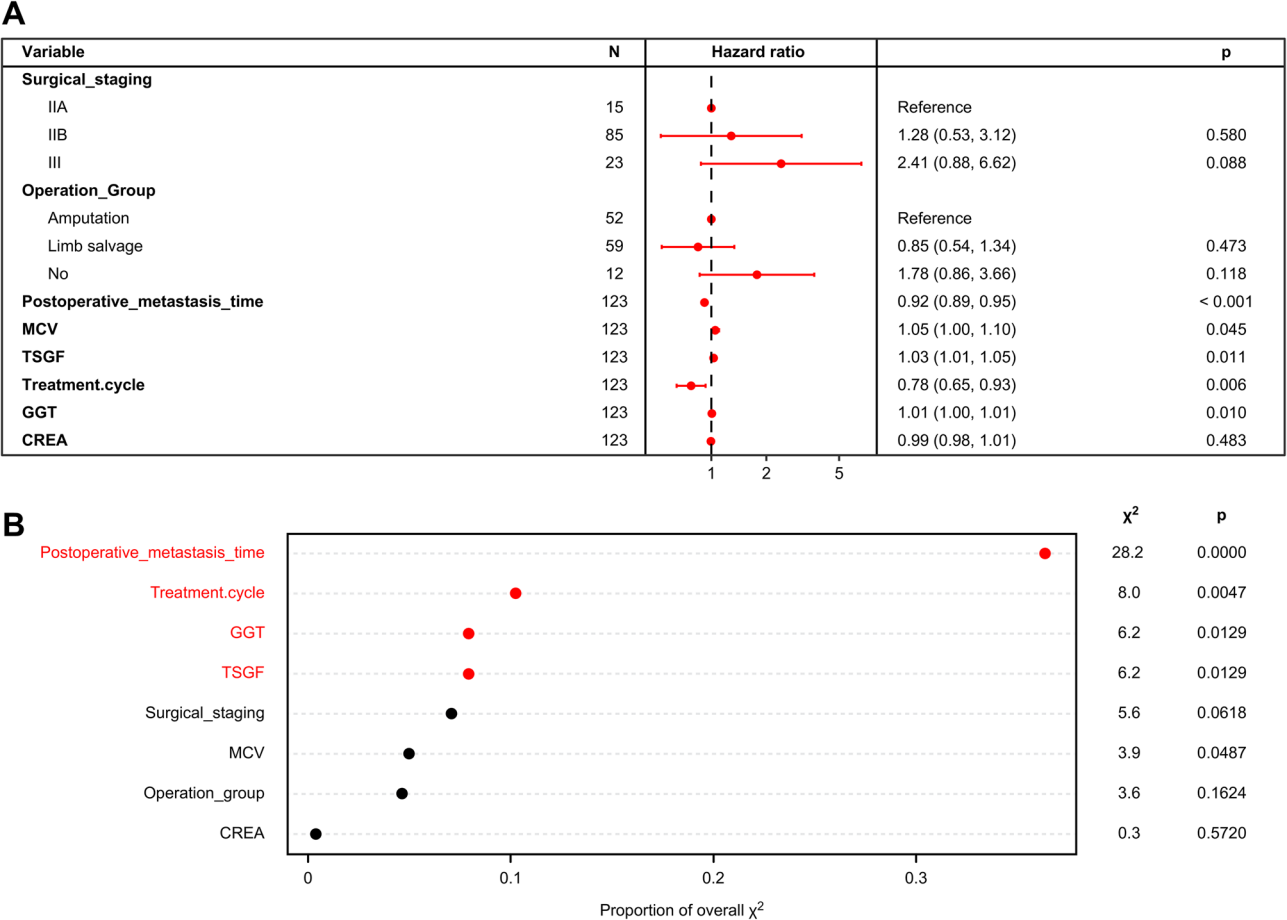
Adjusted analysis of prognostic factors influencing the 5-year overall survival of HOS. (**A**) Forest plot illustrating the hazard ratios for the 5-year overall survival of HOS according to the eight prognostic factors. The dots on the transverse lines represent the hazard ratio (HR), and the transverse lines represent the 95% confidence interval (95% CI). (**B**) Relative contribution of each risk index to the full prediction model.

**Figure 4:**
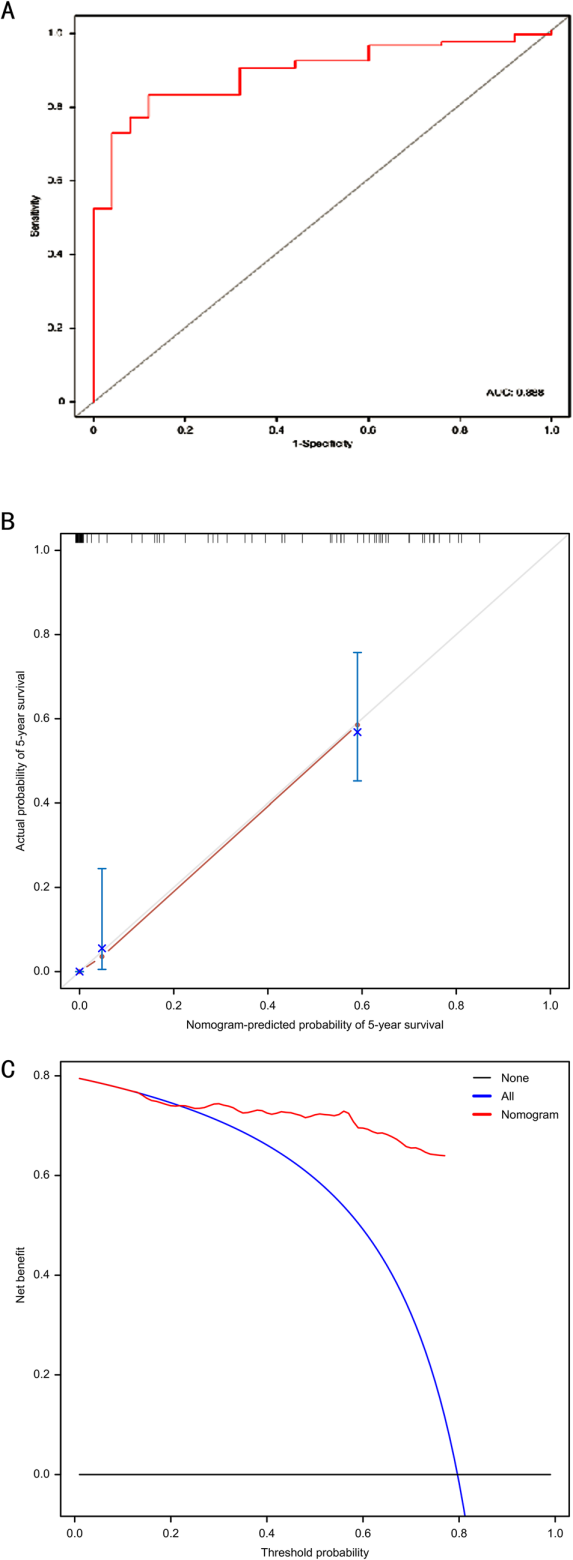
The validation model of the prognostic nomogram. (**A**) ROC analysis of the prognostic nomogram model based on risk factors, including tumour stage, surgical type, metastasis, complete preoperative neoadjuvant chemotherapy cycle, postoperative metastasis time, mean corpuscular volume, tumour-specific growth factor, gamma-glutamyl transferase and creatinine. The area under the receiver operating characteristic (ROC) curve was 0.888. (**B**). Calibration curve for predicting overall survival rate by the nomogram for HOS patients. The y-axis indicates the actual probability of survival; the x-axis indicates the predicted probability of survival by nomogram. The 45-degree grey line represents the ideal prediction; the red line represents the performance of the prognostic nomogram. As the red line approaches the ideal prediction line, the predictive accuracy of the nomogram increases. (**C**). The decision curve analysis curve of the prognostic nomogram. The y-axis indicates the net benefit; the x-axis indicates the threshold probability. The black line represents the hypothesis that no patients die within 5 years; the blue line represents the hypothesis that all patients die within 5 years; the red line represents the prognostic nomogram. *DCA* decision curve analysis.

The univariate survival analysis, a total of 16 factors, including tumour stage, metastasis, complete preoperative chemotherapy cycle, type of surgical, TSGF, MCHC, ALP, LDH, serum Mg, postoperative metastasis time, CA724, GGT, CREA, MCV, BASO and RDW-SD, were statistically associated with the mortality of HOS. In the multivariate model, we found that only the tumour stage, metastasis, complete preoperative chemotherapy cycle, postoperative metastasis time, TSGF, CA724, GGT, MCV and RDW-SD were directly and independently linked to the HOS-related survival time. To formulate an optimal nomogram model, the CA724 and RDW-SD were removed from the model because of their relatively small C-index. Therefore, a nomogram containing tumour stage, surgical type, postoperative metastasis time, MCV, TSGF, treatment cycle, GGT, and CREA showed a better prognostic accuracy in OS than other models (Fig. 2). The associated internal validation C-index for 5-year survival was 0.795 (0.703–0.887). We also tested the proportionality of hazards over the follow-up time. The overall test was not statistically significant (*p* = 0.680). The bias-corrected concordance index of the prognostic nomogram for 5-year OS was 0.708 in the internal validation. According to the forest plot (Fig. 3A) and relative contribution curve (Fig. 3B), the postoperative metastasis time, preoperative neoadjuvant chemotherapy cycle, TSGF and GGT contributed greatly to the prognosis, while surgical staging, MCV, operation type and CREA showed a minor impact on the outcome. The associated AUC in the ROC analysis was as high as 0.888 (Fig. 4A). Otherwise, the calibration plots for the probability of 5-year OS showed a better consistency between the prediction by the nomogram and the real observed outcome (Fig. 4B). Figure 4C illustrates the decision curve analyses of the prediction model to discriminate survivors and non-survivors at the time point of 5-year OS, showing the large net benefits of the model for predicting 5-year survival. This superior performance of the nomogram indicated that it is a better predictive model for OS in patients with HOS.

## Discussion

In recent decades, the clinical treatment of HOS has entered the plateau stage, with poor therapeutic effects and poor prognosis, especially for those with metastases. There have been some studies on the clinical prognosis of HOS, but due to a single evaluation factor or the small sample size, the clinical application was affected. To improve the survival rate, we comprehensively evaluated the potential prognostic valuation of some routine laboratory variables for the OS of HOS and successfully developed an optimal prognostic nomogram model to predict the 5-year OS. To our knowledge, this is the first study to develop a nomogram model to predict the survival probability of Chinese HOS patients using the most comprehensive baseline laboratory testing indicators.

In our cohort, the 5-year OS was 20.3%, while for the non-metastasis patients, the 5-year OS was 52.1%. The survival rates of the non-metastasis patients in China were generally consistent with those reported in other countries. In 2012, the European intergroup osteosarcoma study reported that the 5-year OS was 56% in 1067 localized HOS patients younger than 40 years old. In 2001, the Rizzoli Institute identified that the 8-year survival rate was 59% for 300 non-metastatic osteosarcoma patients^21^. In the past 30 years. The survival rate of HOS patients in China has improved, especially for patients without distant metastasis^4^. The prognosis of patients with metastasis is relatively poor in the later stage. Overall survival for all subjects was slightly lower than in other studies, probably because of the few patients without distant metastases in our study. Patients with distant metastases are the current treatment challenge, as in other countries. In our study, the metastasis rate was 61%. Of these, 34.7% had metastasized at the time of first diagnosis, and 62.7% had metastases within 2 years after diagnosis, which was slightly higher than the values reported by other investigators. Thus, this finding reflected that there was a high proportion of patients with microscopic tumour spread at diagnosis among our patient population. A previous study suggested that patients with metastasis at initial diagnosis and multiple modules were associated with poorer prognosis than patients with solitary metastatic nodules. Eleuterio et al.^22^. reported that the 5-year OS of metastatic and non-metastatic children was 11.9% versus 57.4%, respectively. The above data analysis showed that compared with patients in other countries, the tumour-related survival of Chinese HOS patients was comparable, but the rate of metastasis was relatively higher. Based on these findings, we believe that distant metastasis is one of the strongest risk factors for poor clinical prognosis in Chinese HOS patients. It is important to explore the mechanism of metastasis.

Routine laboratory markers have been widely used to predict clinical survival and long-term prognosis in various cancers. It is noteworthy that, for the first time, we systematically analysed the relationship of 17 haematological variables and 48 serum biochemistry variables with the outcome of HOS patients. We confirmed that nine risk factors were significantly related to poor survival of HOS, including tumour stage, surgical strategy, metastasis, postoperative metastasis time, failure to complete preoperative neoadjuvant chemotherapy, TSGF, MCV, GGT and CREA. In the clinic, elevated serum levels of ALP and LDH are used by surgeons in the diagnosis of osteosarcoma. In our results, serum ALP and LDH were found to be of significance importance in the univariate analysis, but they lost significance after multivariate analysis. The possible cause was that risk factors such as serum TSGF, MCV, GGT and CREA were more effective in predicting mortality than serum ALP and LDH.

Consistent with many previous reports, our study of the Chinese population also demonstrated that tumour stage, metastasis, failure to complete preoperative neoadjuvant chemotherapy, and postoperative metastasis time were associated with poor outcomes in HOS patients. Undoubtedly, the main reasons for poor prognosis were large tumour stage, distant metastasis at the time of diagnosis, and postoperative metastasis time. Some articles have definitively shown that standard preoperative Adriamycin-cisplatin (AP) chemotherapy is an effective treatment for patients with osteosarcoma^10^. In our study, we demonstrated that completed preoperative neoadjuvant chemotherapy cycles have a greater survival benefit than multiagent regimens. As Faisham et al.^23^ reported that the 5-year OS for patients who completed preoperative chemotherapy and surgical treatment was 44%, while it was only 13% for patients who underwent no surgical intervention and did not complete the chemotherapy course. We add evidence that failure to complete preoperative chemotherapy cycles is an adverse prognostic factor for long-term survival in HOS patients.

More remarkably, our findings added evidence that TSGF, GGT, MCV and CREA are favourable prognostic indicators for HOS for the first time. In particular, elevated TSGF levels may be the most paramount prognostic factor for patients with HOS. Previous studies have shown that TSGF is expected to become a molecular marker for the early diagnosis and treatment of small cell lung cancer, breast cancer, gastric cancer, colon cancer and other malignant tumours. A report in 2015 showed that the combined detection of serum TSGF, CEA, CA724 and CA199 can improve the accuracy and sensitivity of gastric cancer diagnosis. A recent study reported that TSGF may be a reliable factor in evaluating the curative effect of neoadjuvant chemotherapy for osteosarcoma^24^. Although there was no study evaluating the prognostic value of MCV, CREA and GGT in patients with osteosarcoma, several studies suggested that these factors may be related to the diagnosis and prognosis of other malignancies. MCV was reported to be a promising molecular marker for the early diagnosis of oesophageal cancer after oesophagectomy^25,26^. In 2019, Jomrich et al. reported that high serum levels of MCV predicted poor outcome for patients with gastroesophageal adenocarcinoma. It has been reported that the preoperative serum level of CREA may be an independent prognostic factor in patients with epithelial ovarian cancer. Recently, many studies have shown that elevated serum GGT levels are a qualified component for the early prediction of cancers. In this study, we stressed that elevated serum GGT levels may be a potential risk factor for the prognosis of HOS patients. In 2016, among 1662,087 Koreans covered by the National Health Insurance Service, 129,087 (7.8%) new cancer cases may have been associated with high serum GGT during 17 years of follow-up. A 2017 study assessed the potential link between GGT and prostate cancer risk A report in the Kuopio prospective cohort for ischaemic heart disease in Finland showed that serum GGT was related to the overall cancer risk. Our findings showed that the combined detection of serum TSGF, GGT, MCV and CREA can improve the prognostic accuracy and sensitivity for the survival of HOS. TSGF contributed greatly to prognostication, while CREA had a minor impact on outcome.

A nomogram is a reliable tool to quantify death risk by evaluating important risk factors for oncologic prognoses. This study built the first nomogram models to accurately predict the survival of HOS patients based on the most clinical laboratory features in China. In this project, we integrated nine factors into the nomogram curve to predict the clinical prognosis of HOS patients and achieved a better predictive ability (AUC = 0.888). Based on the model, we concluded that TSGF, GGT, MCV and CREA were risk factors for poor outcomes in the HOS population, although the precise mechanism should be elucidated in the future. Furthermore, we also observed that TSGF was more important than the other three routine laboratory indicators in the prediction of HOS survival.

Therefore, there are three novel findings of this study. First, this study was the most comprehensive prognostic analysis containing the most clinical laboratory factors. Second, we first reported and carefully evaluated the prognostic value of TSGF, GGT, MCV and CREA in patients with HOS. Third, we constructed a nomogram model facilitating individualized prediction of OS in HOS patients at initial diagnosis. This tool will facilitate physicians in making individualized clinical decisions. Most importantly, our nomogram presented good discriminative ability, with a C statistic of 0.795. However, there are some limitations in this study. First, we explored and validated the nomogram with retrospective data from a single province of China. Multicentre validation with more patients and longer follow-up times is essential for future clinical application. Second, this study does not design external validation sets given the small sample size. A description of the nomogram model from Frank E Harrell is that a nomogram is a graphical device, not a model to validate. However, it is unclear whether this model can be expanded to the entire HOS patient population without external validation. Therefore, external validation is needed in the future to validate the recommended nomogram in a systems biomedical framework^27,28^. Third, this study fails to consider other genomic and clinical risk factors, such as genetic mutations, RNA modifications, immune microenvironment, and nutritional status^29–34^. The next step will be incorporating these factors and assessing the causal effects of multiple factors on HOS by mendelian randomization analysis^35–37^ and try to use key factors to predict HOS by using deep learning models^38^.

In conclusion, the prognosis of Chinese HOS patients has improved and is comparable with that of patients from other countries. Based on a large, population-based cohort, we developed a comprehensive prognostic evaluation system that predicts the 5-year OS of HOS patients. This system proved reliable for risk quantification in HOS patients. Using this predictive tool, clinical doctors can precisely estimate the 5-year OS of individual patients to make precise individualized treatment decisions.

## Methods

### Patients and treatments

**Figure 5:**
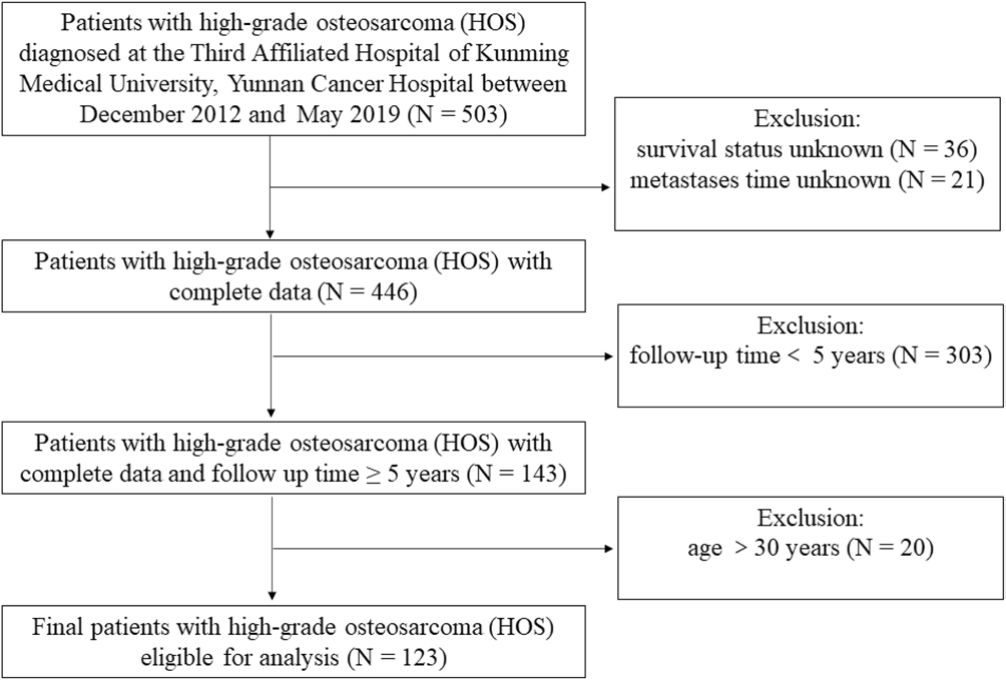
Flow diagram of patients.

This was a retrospective cohort study of some patients diagnosed with HOS from December 2012 to May 2019 in the Third Affiliated Hospital of Kunming Medical University, Yunnan Cancer Hospital, Yunnan, China. It was approved centrally by the ethics committee of the Third Affiliated Hospital of Kunming Medical University, Yunnan Cancer Hospital. This study was carried out according to the Declaration of Helsinki. Prior to the implementation of the study, we signed informed consent forms with all study patients or their legal representatives. The inclusion criteria were as follows: (a) typical radiographic and histologic features of primary HOS by multidisciplinary experts; (b) no previous history of cancer and no prior treatments; (c) age of 30 years or younger; (d) no chemotherapy complications; (e) patients given neoadjuvant chemotherapy; (f) minimal follow-up of 5 years after diagnosis of HOS unless deceased; (g) known cause of death; and (h) complete laboratory blood tests. The exclusion criteria included the following: (a) patients suffering from other cancers simultaneously; (b) age older than 30 years; (c) missing survival status data or laboratory results; and (d) loss to follow-up. In accordance with the inclusion and exclusion criteria, we excluded the data of 380 patients. A total of 123 patients with HOS were enrolled in our study. Patients were excluded for the following reasons (Fig. 5): incomplete data (n = 57); follow-up for fewer than 5 years (n = 303); and age older than 30 years (n = 20). Routine haematological and biochemical detection, normal renal, hepatic, magnetic resonance imaging of HOS, whole-body bone scintigraphy and chest computed tomography (CT) and magnetic resonance imaging (MRI) were performed for all patients at the time of initial diagnosis. The final diagnosis and classification were determined by several senior orthopaedic surgeons after evaluating imaging and pathological diagnosis reports on the basis of the ennecking classification. Routinely, definitive surgery was performed after two cycles of adjuvant chemotherapy. First-line chemotherapy was a combination of doxorubicin (DDP) 60–90 mg/m^2^ per course and cisplatin (ADM) 60–80 mg/m^2^ per course. Additional chemotherapeutic drugs, such as high-dose methotrexate (MTX) 8–12 g/m^2^ per course, vincristine (VCR) 2 mg/m^2^ per course or ifosfamide (IFO) 6–10 g/m^2^ per course, were added to some patients. In our study, the preoperative neoadjuvant chemotherapy cycle was divided into 0–8 cycles, in which a chemotherapy cycle of more than 2 cycles was defined as “complete preoperative chemotherapy”, and a chemotherapy cycle of less than 2 cycles was defined as “failure to complete preoperative chemotherapy”. Follow-ups were performed on the following schedule: once every 3 months in the first 2 years, once every 4 months in the third year, once every 6 months in the 4th and 5th years, and once a year after the 5th year. We ascertained the survival status through telephone interviews.

### Clinical prognostic factors

Data concerning demographics and laboratory testing features of patients were retrospectively collected from the clinical patient database of the Third Affiliated Hospital of Kunming Medical University, Yunnan Cancer Hospital (Kunming, Yunnan, China) and were reviewed by a panel. Two clinicians used the Excel database to screen, confirm, input and sort out the patient data. To ensure the accuracy of the data, we selected two senior doctors to conduct spot checks and evaluate the data. The data must be rechecked and corrected by two chief physicians with more than 10 years of clinical experience. Clinical demographics and laboratory features were as follows: sex, age at diagnosis, ethnicity, height, weight, body mass index (BMI), histology, primary tumour site, tumour stage, tumour grade, maximal tumour size, presence or absence of pathological fracture at diagnosis, time from onset of symptoms to diagnosis, chemotherapy regimens, biopsy method, type of surgery, presence or absence of metastases, tumour recurrence after operation, survival state, follow-up time, tumour necrosis rate after chemotherapy, 17 haematological indexes and 48 serum biochemistry indexes at diagnosis. The levels of 17 haematological indexes were determined by the Coulter principle in all patients, including white blood cells (WBCs), neutrophils (NEUTs), basophilic granulocytes (BASOs), eosinophils (EOs), lymphocytes (LYMPHs), monocytes (MONO), red blood cells (RBCs), haematocrit (HCT), mean corpuscular volume (MCV), mean corpuscular haemoglobin (MCH), mean corpuscular haemoglobin concentration (MCHC), red blood cell volume distribution width (RDW-SD), red blood cell volume coefficient of variation (RDW-CV), mean platelet volume (MPV), thrombocytocrit (PCT-L), platelet distribution width (PDW), and platelet-large cell ratio (P-LCR). 48 serum laboratory biochemical indicators are as follows: total protein (TP), albumin (ALB), albumin/globulin ratio (A/G), adenosine deaminase (ADA), alpha-L-fucosidase (AFU), alkaline phosphatase (ALP), Alanine transaminase (ALT), aspartate aminotransferase (AST), blood urea nitrogen (BUN), calcium (CA), cholinesterase (CHE), chlorine (CL), carbon dioxide (CO_2_), creatinine (CREA), direct bilirubin (D-BIL), serum iron (Fe), gamma-glutamyl transferase (GGT), globulin (GLO), glucose (GLU), indirect bilirubin (IBIL), potassium (K), lactic acid dehydrogenase (LDH), magnesium (Mg), serum sodium (Na), osmotic pressure (Osm), serum phosphorus (P), prealbumin (PA), total bile acids (TBA), total bilirubin (TBIL), uric acid (UA), prothrombin time international normalized ratio (PT-INR), prothrombin time ratio (PT-R), Prothrombin time (PT-SEC), Prothrombin time (TT), alpha-foetoprotein (AFP), carcinoembryonic antigen (CEA), squamous cell carcinoma antigen (SCC), carbohydrate antigen 19-9 (CA19-9), carbohydrate antigen 242 (CA242), carbohydrate antigen 724 (CA724), neuron-specific enolase (NSE), carbohydrate antigen 125 (CA125), carbohydrate antigen 15-3 (CA15-3), ferritin (Ferr), cytokeratin 19 fragment (CYFRA21-1), tumour-specific growth factor (TSGF), plasma fibrinogen (FIB), activated partial thromboplastin time (APTT). Routine blood biochemical examination was applied using the Cobas 8000 automatic biochemical analyser (Roche, USA). Other blood testing indexes were determined using the System XE-2100 automatic blood cell analyser (SYSMEX corporation, Japan). Considering that HOS mainly occurs in adolescents, we mainly focused on adolescents younger than 30 years of age in this study. The age at diagnosis was categorized into two groups: ≤ 18 years old and 18–30 years. Tumour primary sites were stratified into five groups: femur, tibia, humerus, fibula, and other. Tumour size was divided into two groups: < 10.0 cm and ≥ 10.0 cm. Tumour stage was divided into three groups: stage IIA (non-metastatic cases), stage IIB (non-metastatic cases) and stage III (primary metastatic cases). The treatment regimens were divided into three groups: ADM/DDP, ADM/DDP/MTX/VCR and ADM/DDP/MTX/VCR/IFO. The treatment cycle was divided into two groups, and the patients who received at least two cycles of preoperative neoadjuvant chemotherapy were the complete treatment cycle. According to the cut-off value, the quantitative variables were dichotomized into the normal group (< cut-off value) and the elevated group (≥ cut-off value). The optimal cut-off scores were established based on the log-rank statistics of different factors.

### Risk feature selection

The main aim of the present study was to determine the 5-year OS of HOS patients under 30 years of age and to identify the demographic and clinicopathologic covariates for OS. OS was defined as the length of time from the date of diagnosis to the date of the last follow-up or death from any cause. Censored observations referred to patients alive at the date of last follow-up. The optimal cut-off of the continuous variables provided the largest disparity in OS between the high- and low-risk groups on the basis of the log-rank statistic. For each covariable, all patients were then dichotomized into two groups based on the optimal cut-off points. The optimal cut-off values of the continuous variables were selected using the maximally selected log-rank statistics from the maxstat package. Any missing variable values were assumed to be missing at random, and their values were imputed with random forest missing data algorithms implemented in the randomforestSRC package^39^. Survival curves for different variable values were depicted according to the Kaplan–Meier method and compared across the levels of variables using the log-rank test. The Kaplan–Meier survival curves were plotted using the survminer package. Univariate and multivariate analyses of overall survival were used to examine variables using the Cox proportional hazards regression model. Variables that were statistically significant at *p* < 0.05 in univariate analysis were included in the multivariable analyses by using the survival package. In addition, given that this study was exploratory data analysis, no adjustments for multiple comparisons were conducted. A penalized Cox proportional hazards regression model using the least absolute shrinkage and selection operator (LASSO) penalty, which is suitable for the regression of high-dimensional data, was applied to select the best variables for the nomogram of OS. Penalty parameter tuning in the LASSO Cox proportional hazards regression model was conducted by 100 iterations of tenfold cross-validation (CV) with minimum criteria using the glmnet package, and then the variables with a non-zero Cox regression coefficient were selected as the important prognostic factors. The Schoenfeld residual test was used to test the proportional hazards assumption for all variables included in the Cox proportional hazards regression model by using the survival and survminer package^40^, and metastatic status was found to be non-proportional. The subsequent model was stratified by metastatic status to satisfy the proportional hazards assumption. The hazard ratios (HRs) and the associated 95% confidence intervals (CIs) were estimated for each variable included in the stratified Cox proportional hazards model while adjusting for other covariates. The relative contribution of each predictor to the stratified Cox proportional hazards model was compared using the Wald chi-square statistics computed by the rms package.

### Nomogram development and internal validation

The performance of the stratified Cox proportional hazards model was evaluated by calculating Harrell's concordance index (C-index), which is the area under the receiver operating characteristic (ROC) curve adapted for censored data with the use of the survival package^41–43^. The value of the C-index ranges from 0.5 to 1.0. C = 0.5 indicates random discrimination. C = 1.0 indicates perfect predictive accuracy. A model with a larger C-index was considered to have a more accurate prediction. The survival ROC curves were plotted using the survivalROC package. On the basis of the stratified Cox proportional hazards model with identified prognostic factors, a prognostic nomogram for predicting the 5-year survival rates of HOS patients was developed with the rms package. The prognostic nomogram was validated by measuring discrimination and calibration curves with the use of the rms package. To gauge the discrimination performance of the prognostic nomogram, we calculated the bias-corrected concordance index using the bootstrap method in the original dataset by 1000 re-samplings to correct for potential overfitting bias. We constructed a calibration curve to assess whether the predicted survival probabilities and the actual outcome were in concordance. Bootstraps with 1000 resamples were used to compare concordance probabilities. The more similar the calibration curve along the 45-degree line, the better calibrated the prognostic prediction. The clinical usefulness of the prognostic nomogram was evaluated using decision curve analysis (DCA). DCA was performed by calculating the net benefit of nomogram-assisted decisions across a range of threshold probabilities^44^. The decision curve was plotted using the dca package.

### Statistical analysis

All statistical analyses were conducted using SPSS Statistics version 25.0 (IBM Corporation, Armonk, NY, USA) and R statistical software version 3.2.4. All statistical tests were two-sided, and a *p* value < 0.05 was deemed statistically significant. Continuous variables with a normal distribution are presented as the mean ± standard deviation (${\overline{\text{X}}}$ ± S), and categorical variables are presented as frequencies (percentages). Student’s *t* tests, chi-square tests and nonparametric Mann–Whitney U tests were conducted to determine the differences between two groups.

### Ethical approval

All methods were carried out in accordance with relevant guidelines and guidelines and regulations.

## Supplementary Information

Supplementary Table S1.

Supplementary Table S2.

Supplementary Table S3.

## Data Availability

The data that support the findings of this study are available from the corresponding author upon reasonable request.
