# Author Correction: Prognostic nomogram for predicting 5-year overall survival in Chinese patients with high-grade osteosarcoma

**DOI:** 10.1038/s41598-021-98442-6

**Published:** 2021-09-15

**Authors:** Zhihong Yao, Zunxian Tan, Jifei Yang, Yihao Yang, Cao Wang, Jiaxiang Chen, Yanan Zhu, Tiying Wang, Lei Han, Lin Zhu, Zuozhang Yang

**Affiliations:** 1 Bone and Soft Tissue Tumours Research Centre of Yunnan Province, Department of Orthopaedics, The Third Affiliated Hospital of Kunming Medical University (Yunnan Cancer Hospital), Kunming, 650118 Yunnan 119 Kunzhou Road, China; 2 Independent Investigator, No. 22 Xi guan North Road, Kaifeng, 475000 Henan China

Correction to: *Scientific Reports* 10.1038/s41598-021-97090-0, published online 06 September 2021.

In the original version of this Article, Lin Zhu was omitted as a corresponding author. Correspondence and requests for materials should also be addressed to zhulinkin@foxmail.com.

The original Article has been corrected.

