# Supplementary material for: Prognostic nomogram for predicting 5-year overall survival in Chinese patients with high-grade osteosarcoma
Source: Sci Rep. 2021 Sep 6;11:17728. doi: 10.1038/s41598-021-97090-0 (PMC8421452; doi:10.1038/s41598-021-97090-0)
Supplement: Supplementary file 1 — Supplementary Table S1. [file 41598_2021_97090_MOESM1_ESM.docx]

# Supplementary table S1 Baseline clinical characteristics of HOS patients in the alive group and death group with with continuous variables

| **Variables** | **Alive no. cases (%)**  **(N = 25)** | **Death no. cases (%)**  **(N = 98)** | **Total** | ***p*** |
| --- | --- | --- | --- | --- |
| Age, years | 16.04±4.74 | 16.80±5.36 | 16.64± 5.23 | 0.521 |
| BMI, kg/m^2^ | 18.90±3.39 | 18.68±3.39 | 18.72± 3.37 | 0.767 |
| WBC, 10^9^/L | 7.47±2.23 | 7.50±2.33 | 7.49±2.31 | 0.953 |
| NEUT, 10^9^/L | 4.78±2.00 | 4.65±2.00 | 4.67±1.93 | 0.768 |
| EO, 10^9^/L | 0.16±0.13 | 0.16±0.20 | 0.163±0.18 | 0.89 |
| LYMPH, 10^9^/L | 2.09±0.55 | 2.23±0.71 | 2.20±0.68 | 0.346 |
| MONO, 10^9^/L | 0.44±0.15 | 0.44±0.19 | 0.44±0.18 | 0.939 |
| RBC, 10^9^/L | 5.14±0.37 | 5.02±0.55 | 5.04±0.52 | 0.189 |
| HCT, % | 43.59±4.19 | 43.25±5.01 | 43.31±4.84 | 0.758 |
| MCV, fL | 84.80±4.84 | 86.53±4.86 | 86.18±4.89 | 0.114 |
| MCH, pg | 29.05±1.87 | 28.96±1.85 | 28.98±1.84 | 0.829 |
| RDW.CV, % | 12.90±0.64 | 13.15±1.22 | 13.1±1.12 | 0.148 |
| MPV, fL | 10.25±0.92 | 10.54±1.00 | 10.48±0.98 | 0.197 |
| PCT.L, % | 0.32±0.07 | 0.32±0.09 | 0.32±0.09 | 0.932 |
| PDW, fL | 11.64±1.68 | 12.34±2.08 | 12.20±2.02 | 0.124 |
| P.LCR, % | 26.36±7.80 | 28.94±8.23 | 28.41±8.18 | 0.16 |
| PCT.L | 315.84±81.73 | 305.28±99.99 | 307.43±96.34 | 0.627 |
| TP, g/L | 73.73±5.0 | 74.74±5.49 | 74.54±5.39 | 0.402 |
| ALB, g/L | 44.97±3.78 | 45.38±4.59 | 45.30±4.43 | 0.679 |
| A.G | 1.61±0.31 | 1.60±0.31 | 1.60±0.31 | 0.915 |
| ADA, U/L | 11.28±3.12 | 10.78±3.52 | 10.88±3.44 | 0.515 |
| AFU, U/L | 25.84±8.32 | 26.65±8.76 | 26.49±8.65 | 0.677 |
| ALT, U/L | 17.92±12.25 | 21.8±21.82 | 21.02±20.26 | 0.242 |
| AST, U/L | 20.48±6.44 | 21.90±8.97 | 21.61±8.51 | 0.459 |
| AST.ALT | 1.40±0.52 | 1.36±0.62 | 1.37±0.60 | 0.756 |
| BUN, mmol/L | 4.83±1.38 | 4.9240±1.4786 | 4.91±1.45 | 0.778 |
| CA, mmol/L | 2.44±0.12 | 2.4328±0.1177 | 2.43±0.12 | 0.748 |
| CHE, U/L | 8396.68±1546.50 | 8233.62±1819.55 | 8266.76± 1762.71 | 0.681 |
| CL, mmol/L | 102.88±2.35 | 101.8061±2.8885 | 102.02±2.81 | 0.088 |
| CO_2_, mmol/L | 23.36±2.77 | 23.6633±2.5116 | 23.60±2.56 | 0.599 |
| CREA, μmol/L | 68.00±17.5285 | 63.5510±15.6054 | 64.46±16.04 | 0.217 |
| D.BIL, μmol/L | 3.6840±1.5253 | 4.1020±1.6565 | 4.017±1.63 | 0.255 |
| Serum Fe, μmol/L | 11.5160±6.9114 | 12.8561±6.2441 | 12.58±6.38 | 0.351 |
| GGT, U/L | 25.1600±24.7785 | 31.8469±65.5233 | 30.49±59.51 | 0.421 |
| GLO, g/L | 28.7600±4.5060 | 29.2316±4.2781 | 29.14±4.31 | 0.627 |
| GLU, mmol/L | 4.7944±0.4245 | 4.8759±0.5276 | 4.86±0.51 | 0.476 |
| IBIL, μmol/L | 5.6240±3.4869 | 6.13±3.64 | 6.03±3.60 | 0.534 |
| K, mmol/L | 4.5100±0.4530 | 4.44±0.36 | 4.45±0.38 | 0.395 |
| Na, mmol/L | 140.8400±1.9723 | 140.26±2.37 | 140.37±2.30 | 0.258 |
| Osm, MoSM | 275.7200±3.5534 | 274.84±4.35 | 275.02±4.20 | 0.35 |
| P, mmol/L | 1.5800±0.2773 | 1.51±0.27 | 1.52±0.27 | 0.235 |
| PA mg/L | 229.120±57.9748 | 231.22±65.72 | 230.80±64.00 | 0.884 |
| TBA, μmol/L | 4.9680±2.5413 | 4.76±3.87 | 4.80±3.63 | 0.748 |
| TBIL, μmol/L | 9.3080±4.9286 | 10.23±5.06 | 10.04±5.03 | 0.416 |
| UA, μmol/L | 363.000±54.7601 | 365.92±91.97 | 365.33±85.54 | 0.84 |
| PT.INR | 1.0252±0.1034 | 1.03±0.08 | 1.03±0.09 | 0.973 |
| PT. RATIO | 1.0184±0.0787 | 1.02±0.06 | 1.02±0.07 | 0.931 |
| PT.SEC | 13.3320±1.1324 | 13.44±0.84 | 13.42±0.90 | 0.649 |
| TT, sec | 16.3840±1.3206 | 16.31±1.21 | 16.33±1.23 | 0.794 |
| AFP, μg/L | 1.8156±0.9910 | 1.89±1.83 | 1.87±1.67 | 0.799 |
| CEA, μg/L | 1.3680±0.8108 | 1.53±1.38 | 1.50±1.28 | 0.454 |
| SCC, μg/L | 0.7925±0.4171 | 0.95±0.50 | 0.92±0.48 | 0.23 |
| CA.199, kU/L | 11.2373±10.4229 | 11.10±9.95 | 11.11±9.97 | 0.959 |
| CA.242, kU/L | 8.0219±6.9166 | 9.93±8.69 | 9.55±8.36 | 0.418 |
| CA.724, kU/L | 2.2044±1.8925 | 3.12±4.31 | 2.94±3.94 | 0.208 |
| NSE, μg/L | 26.1447±34.9776 | 32.60±48.04 | 31.35±45.70 | 0.604 |
| CA.125, kU/L | 13.2533±7.4661 | 16.62±15.61 | 15.96±14.39 | 0.285 |
| CA.153, kU/L | 12.6090±5.9343 | 14.25±11.81 | 13.98±11.01 | 0.52 |
| Ferr, μg/L | 217.460± 179.822 | 243.92±197.74 | 239.14±193.84 | 0.635 |
| CYFRA21-1, μg/L | 1.9786±0.5177 | 4.77±21.45 | 4.3094±19.61 | 0.277 |
| FIB, g/L | 3.9752±1.4923 | 4.18±1.36 | 4.13±1.39 | 0.521 |
| APTT, Sec | 42.3760±5.1567 | 40.90±7.33 | 41.20±6.95 | 0.346 |
| MCHC, g/L | 342.6±11.4 | 335.1±13.4 | 336.6±13.3 | 0.011 |
| RDW.SD, fL | 39.6±2.6 | 41.1±3.4 | 40.8±3.3 | 0.039 |
| ALP, U/L | 237.7±214.0 | 499.4±811.4 | 446.2±737.3 | 0.005 |
| LDH, U/L | 208.3±111.9 | 280.4±270.9 | 265.8±248.4 | 0.044 |
| Serum Mg, mmol/L | 0.8±0.03 | 0.8±0.06 | 0.8±0.06 | 0.001 |
| TSGF, U/mL | 42.5±11.1 | 51.5±11.8 | 49.6±12.1 | 0.003 |
| CREA, μmol/L | 68.0±17.5 | 63.6±15.6 | 64.5±16.0 | 0.217 |
| GGT, U/L | 55.2±111.1 | 23.8±37.6 | 31.9±65.5 | 0.177 |
| MCV, fL | 86.1±5.1 | 86.7±4.8 | 86.5±4.9 | 0.629 |
