# Supplementary material for: Prognostic nomogram for predicting 5-year overall survival in Chinese patients with high-grade osteosarcoma
Source: Sci Rep. 2021 Sep 6;11:17728. doi: 10.1038/s41598-021-97090-0 (PMC8421452; doi:10.1038/s41598-021-97090-0)
Supplement: Supplementary file 2 — Supplementary Table S2. [file 41598_2021_97090_MOESM2_ESM.docx]

##### Table S2 Baseline demographics and clinical characteristics of HOS patients in the metastasis group and non-metastasis group

| Variables | Metastasis group  (N = 75) | Non-metastasis group  (N = 48) | *p* |
| --- | --- | --- | --- |
| Age (y) |  |  | 0.576 |
| ≥18 | 29 (38.67) | 21 (43.75) |  |
| <18 | 46 (61.33) | 27 (56.25) |  |
| Gender |  |  | 0.474 |
| Male | 56 (74.67) | 33 (68.75) |  |
| Female | 19 (25.33) | 15 (31.25) |  |
| Race |  |  | 0.494 |
| Han | 46 (61.33) | 27 (56.25) |  |
| Minority | 29 (38.67) | 21 (43.75) |  |
| BMI group | | | 0.071 |
| <22 | 68 (90.67) | 38 (79.17) |  |
| ≥22 | 7 (9.33) | 10 (20.83) |  |
| Tumour site group | | | 0.732 |
| Femur | 39 (52) | 24 (50) |  |
| Fibula | 4 (5.33) | 4 (8.33) |  |
| Humerus | 2 (2.67) | 3 (6.25) |  |
| Tibia | 24 (32) | 15 (31.25) |  |
| Other | 6 (8) | 2 (4.167) |  |
| Tumour stage | | | <0.001 |
| IIA | 6 (8) | 9 (18.75) |  |
| IIB | 47 (62.67) | 38 (79.17) |  |
| III | 22 (29.33) | 1 (2.08) |  |
| Tumour size | | | 0.066 |
| <10 | 28 (37.33) | 26 (54.17) |  |
| ≥10 | 47 (62.67) | 22 (45.83) |  |
| Pathological fracture | | | 0.371 |
| Yes | 7 (9.33) | 7 (14.58) |  |
| No | 68 (90.67) | 41 (85.42) |  |
| Treatment group | | | 0.964 |
| ADM/NDP | 16 (21.33) | 9 (18.75) |  |
| ADM/DDP/MTX/VCR | 8 (10.67) | 6 (12.50) |  |
| ADM/DDP/MTX/VCR/IFO | 47 (62.67) | 31 (64.58) |  |
| No | 4 (5.33) | 2 (4.167) |  |
| Operation group | | | 0.979 |
| Amputation | 32 (42.67) | 20 (41.67) |  |
| Limb salvage | 36 (48.00) | 23 (47.92) |  |
| No | 7 (9.33) | 5 (10.42) |  |
| Survival state group | | | <0.001 |
| No | 2 (2.67) | 23 (47.92) |  |
| Yes | 73 (97.33) | 25 (52.08) |  |
| Recurrence | | | 0.138 |
| Yes | 9 (12) | 2 (4.17) |  |
| No | 66 (88) | 46 (95.83) |  |
| Complete the treatment cycle | | | 0.373 |
| Yes | 44 (58.67) | 32 (66.67) |  |
| No | 31 (41.33) | 16 (33.33) |  |
| TSGF group (cut off = 54±3.0, U/mL) | | | 0.882 |
| normal | 40 (67.80) | 27 (69.23) |  |
| elevated | 19 (32.2) | 12 (30.77) |  |
| GGT (cut off = 113±1.8, U/L) | | | 0.838 |
| normal | 73 (97.22) | 47 (97.92) |  |
| elevated | 2 (2.67) | 1(2.08） |  |
| MCV (cut off = 90.1±2.7, fL) | | | 0.420 |
| normal | 58 (77.33) | 40 (83.33) |  |
| elevated | 17 (22.67) | 8 (16.67) |  |
| CREA (cut off = 68±3.1, μmol/L) | | | 0.216 |
| normal | 49 (65.33) | 26 (54.17) |  |
| elevated | 26 (34.67) | 22 (45.83) |  |
| LDH (cut off = 185±3.8, U/L) | | | 0.004 |
| normal | 20 (26.67) | 25 (50.08) |  |
| elevated | 55 (73.33) | 23 (47.2) |  |
| ALP (cut off = 343±2.6, U/L) | | | 0.006 |
| normal | 49 (65.33) | 42 (87.5) |  |
| elevated | 26 (34.67) | 6 (12.5) |  |

*Abbreviations:* Other variables included ADM/DDP/MTX, ADM/IFO, ADM/DDP/MTX/IFO, and ADM/IFO. ALP, alkaline phosphatase. The cut-off value was determined based on receiver operating characteristic curve analysis (ROC).
