# Supplementary material for: Prognostic nomogram for predicting 5-year overall survival in Chinese patients with high-grade osteosarcoma
Source: Sci Rep. 2021 Sep 6;11:17728. doi: 10.1038/s41598-021-97090-0 (PMC8421452; doi:10.1038/s41598-021-97090-0)
Supplement: Supplementary file 3 — Supplementary Table S3. [file 41598_2021_97090_MOESM3_ESM.docx]

# Supplementary table S3 Univariate analysis for the risk factors associated with the survival of HOS patients

| Variables | HR (95% CI) | *p* |
| --- | --- | --- |
| WBC | 1.032 (0.952-1.118) | 0.448 |
| NEUT | 1.024 (0.927-1.132) | 0.636 |
| EO | 0.850 (0.318-2.322) | 0.765 |
| LYMPH | 1.197 (0.891-1.607) | 0.233 |
| MONO | 1.363 (0.439-4.233) | 0.592 |
| RBC | 0.703 (0.467-1.060) | 0.093 |
| HGB | 0.993 (0.981-1.004) | 0.210 |
| HCT. | 0.989 (0.946-1.035) | 0.638 |
| RDW.CV | 1.101 (0.917-1.323) | 0.302 |
| MPV | 1.134 (0.921-1.395) | 0.236 |
| PCT.L. | 0.570 (0.064-5.075) | 0.614 |
| PDW | 1.079 (0.979-1.188) | 0.124 |
| P.LCR | 1.016 (0.992-1.042) | 0.199 |
| TP | 1.004 (0.968-1.041) | 0.823 |
| ALB | 0.017 (0.968-1.068) | 0.503 |
| A. G | 1.341 (0.688-2.613) | 0.389 |
| ADA | 0.975 (0.916-1.038) | 0.430 |
| AFU | 0.996 (0.974-1.019) | 0.723 |
| AST.ALT | 1.063 (0.738-1.529) | 0.744 |
| CA | 0.653 (0.098-4.368) | 0.660 |
| CHE | 1.000 (1.000-1.000) | 0.679 |
| D.BIL | 1.047 (0.932-1.176) | 0.435 |
| Fe | 1.015 (0.986-1.045) | 0.320 |
| GLO | 0.989 (0.945-1.035) | 0.645 |
| GLU | 2.347 (0.866-2.095) | 0.186 |
| IBIL | 1.002 (0.950-1.056) | 0.948 |
| K | 0.869 (0.520-1.451) | 0.592 |
| Na | 0.921 (0.841-1.009) | 0.076 |
| Osm | 0.957 (0.910-1.007) | 0.089 |
| P | 0.553 (0.248-1.235) | 0.148 |
| PA | 1.001 (0.998-1.004) | 0.589 |
| TBA | 0.978 (0.922-1.037) | 0.453 |
| TBIL | 1.005 (0.969-1.043) | 0.771 |
| UA | 1.000 (0.997-1.002) | 0.727 |
| PT.INR | 1.029 (0.108-9.818) | 0.980 |
| PT. RATIO | 1.072 (0.056-20.539) | 0.963 |
| PT.SEC | 1.036 (0.836-1.283) | 0.747 |
| TT | 0.971 (0.820-1.149) | 0.729 |
| AFP | 0.980 (0.867-1.108) | 0.747 |
| CEA | 1.069 (0.887-1.288) | 0.482 |
| BACT | 1.000 (1.000-1.000) | 0.302 |
| SCC | 1.149 (0.738-1.789) | 0.539 |
| CA.199 | 1.004 (0.980-1.029) | 0.746 |
| CA.242 | 1.014 (0.986-1.043) | 0.333 |
| NSE | 1.003 (0.998-1.008) | 0.285 |
| CA.125 | 1.010 (0.993-1.028) | 0.259 |
| CA.153 | 1.014 (0.989-1.040) | 0.260 |
| Ferr | 1 (0.999-1.002) | 0.608 |
| CYFRA21.1 | 0.978 (0.790-1.210) | 0.836 |
| FIB | 1.022 (0.895-1.167) | 0.752 |
| APTT | 0.979 (0.941-1.019) | 0.307 |

HR, hazard ratio; CI, confidence interval.
